# Reprogramming Glial Cell Metabolism via a tRNA Fragment Preserves Vision in Retinal Neurodegeneration

**DOI:** 10.1002/mco2.70879

**Published:** 2026-07-22

**Authors:** Yuke Ji, Sha Liu, Ying Zhang, Junya Zhu, Chang Huang, Ya Zhao, Jiao Xia, Wan Mu, Jin Yao, Biao Yan

**Affiliations:** ^1^ Department of Ophthalmology and Optometry The Affiliated Eye Hospital Nanjing Medical University Nanjing China; ^2^ Shenzhen Eye Hospital Jinan University Shenzhen China; ^3^ School of Medicine Southeast University Nanjing China; ^4^ Eye Institute and Department of Ophthalmology Eye and ENT Hospital Fudan University Shanghai China; ^5^ Department of Ophthalmology Shanghai General Hospital Shanghai Jiao Tong University School of Medicine Shanghai China

**Keywords:** glycerophospholipid metabolism, reactive gliosis, retinal ganglion cell, retinal neurodegeneration

## Abstract

Retinal neurodegeneration leads to progressive and irreversible vision loss driven by retinal ganglion cell (RGC) death, yet effective neuroprotective therapies remain lacking. Recent studies suggest that small non‐coding RNAs play key roles in central nervous system injury, but their relevance to retinal neurodegeneration remains incompletely understood. Here, we identify a significant increase in 5ʹtiRNA‐His‐GTG, an ANG‐generated tRNA‐derived fragment, in mouse models of retinal neurodegeneration. Functionally, elevated 5ʹtiRNA‐His‐GTG promotes reactive gliosis and contributes to RGC degeneration through Müller cell‐RGC crosstalk. Conversely, inhibition of 5ʹtiRNA‐His‐GTG attenuates glial activation, preserves RGC survival, and improves visual function and vision‐dependent behaviors. Mechanistically, 5ʹtiRNA‐His‐GTG induces neurodegenerative changes by suppressing the LPCAT1‐mediated phosphatidylcholine (PC) biosynthetic pathway and perturbing glycerophospholipid metabolism. Notably, restoration of LPCAT1 expression or PC levels reverses 5ʹtiRNA‐His‐GTG‐induced neurodegeneration both in vitro and in vivo. These findings uncover a previously unrecognized 5ʹtiRNA‐His‐GTG‐LPCAT1‐PC regulatory pathway that contributes to retinal neurodegeneration. Collectively, our study identifies 5ʹtiRNA‐His‐GTG as a critical mediator of glial‐driven neuroinflammation and neuronal loss, and highlights this signaling axis as a potential therapeutic target for retinal neurodegeneration.

## Graphical abstract

Under oxidative and excitotoxic stress, Müller cell‐derived 5′tiRNA‐His‐GTG contributes to retinal neurodegeneration. Pathological stimuli increase ANG‐dependent production of 5′tiRNA‐His‐GTG, which suppresses LPCAT1 expression and disrupts phosphatidylcholine (PC)‐mediated glycerophospholipid metabolism. This metabolic imbalance promotes reactive gliosis, enhances proinflammatory cytokine release, reduces neurotrophic support, and accelerates RGC loss. Thus, targeting the 5′tiRNA‐His‐GTG‐LPCAT1‐PC axis represents a potential therapeutic strategy for retinal neurodegenerative disease.

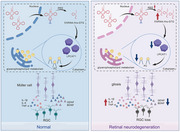

## 1 Introduction

Retinal neurodegenerative diseases, including glaucoma and diabetic retinopathy, are major causes of irreversible blindness worldwide, with glaucoma alone projected to affect over 111 million people by 2040 [1, 2]. Vision loss in these diseases primarily results from the death of retinal ganglion cells (RGCs), the sole neurons that transmit visual information to the brain. Because mature RGCs have little intrinsic regenerative capacity, their loss is largely irreversible [3, 4]. Current treatments mainly focus on managing intraocular pressure and surgical intervention; however, these approaches do not prevent RGC degeneration, highlighting the need for effective neuroprotective therapies [5].

Mounting evidence indicates that glial cells are key components of the retinal microenvironment and play important roles in maintaining neuronal survival. Microglia and macroglia, including Müller cells and astrocytes, support retinal homeostasis by providing neurotrophic factors, regulating metabolic balance, and clearing excess glutamate [6, 7, 8]. Under pathological stress, glial cells undergo reactive gliosis. Although initially protective, prolonged glial activation contributes to RGC degeneration through the release of pro‐inflammatory cytokines, excessive glutamate, and reactive oxygen species (ROS) [2, 9, 10]. Recent single‐cell transcriptomic studies have revealed substantial heterogeneity in glial responses during neurodegeneration. However, the mechanisms underlying the shift from neuroprotective to neurotoxic states remain unclear [11, 12].

Although the mechanisms underlying neurodegeneration remain incompletely understood, increasing evidence suggests that gene regulatory networks play a central role in neuronal maintenance and stress responses. Disruption of these networks can impair cellular homeostasis and lead to neuronal degeneration [13]. Early studies focused mainly on protein‐coding genes, whereas non‐coding RNAs, such as lncRNAs, miRNAs, and circRNAs, have emerged as key regulators of neurodegenerative diseases [14, 15, 16, 17]. More recently, attention has turned to tRNA‐derived small RNAs (tsRNAs), particularly tRNA‐derived stress‐induced RNAs (tiRNAs), as novel players in central nervous system injury [18, 19]. tiRNAs are generated by site‐specific cleavage of mature tRNAs under stress conditions and typically range from 31 to 40 nucleotides in length [20]. These small RNAs can inhibit protein translation, promote apoptosis, and are implicated in neurodegenerative diseases such as Parkinson's disease, amyotrophic lateral sclerosis, and cerebral ischemia [21, 22, 23]. However, their role in retinal neurodegeneration remains largely unexplored.

In this study, we identify 5ʹtiRNA‐His‐GTG as a critical regulator of retinal neurodegeneration. Using mouse models of optic nerve crush (ONC) and NMDA‐induced excitotoxicity, we show that 5ʹtiRNA‐His‐GTG is upregulated in Müller glia under stress conditions. Mechanistically, it promotes reactive gliosis and contributes to RGC loss through disrupted Müller cell‐RGC crosstalk. We further reveal a previously unrecognized link between 5ʹtiRNA‐His‐GTG and lipid metabolism. Specifically, 5ʹtiRNA‐His‐GTG suppresses LPCAT1‐phosphatidylcholine (PC) pathway, thereby promoting glial activation and neurotoxicity. Restoration of LPCAT1 expression or PC levels largely rescues these effects, highlighting a potential therapeutic axis for intervention. Collectively, this study reveals 5ʹtiRNA‐His‐GTG as a critical mediator of glia‐driven neurodegeneration and a promising target for retinal neuroprotection.

## 2 Results

### 2.1 5ʹtiRNA‐His‐GTG Is Upregulated in Retinal Neurodegeneration

**FIGURE 1:**
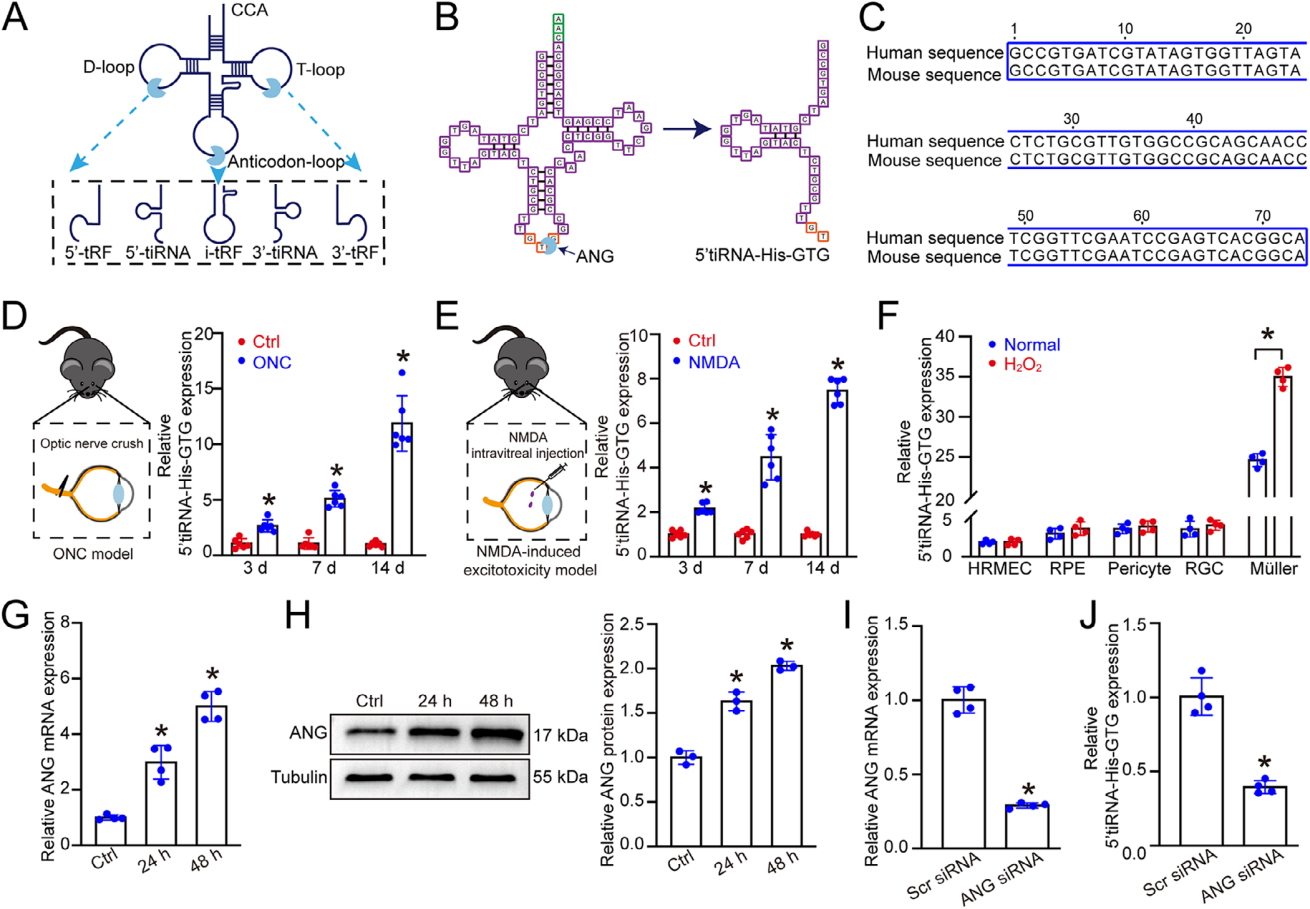
5ʹtiRNA‐His‐GTG is upregulated in retinal neurodegeneration. (A) Schematic overview of tsRNA classification. (B) Illustration of cleavage‐derived production of 5ʹtiRNA‐His‐GTG from mature tRNA‐His‐GTG. (C) Comparative analysis of sequence conservation of 5ʹtiRNA‐His‐GTG between human and mouse. (D, E) Experimental design of ONC model (D) and NMDA‐induced excitotoxicity model (E). Retinas were collected at 3, 7, or 14 days post‐injury, and the expression levels of 5ʹtiRNA‐His‐GTG were detected by qRT‐PCRs (*n* = 6; *p < 0.05, one‐way ANOVA with Bonferroni post hoc test). (F) RGCs, HRMECs, primary Müller cells, pericytes, and RPEs were treated with H_2_O_2_ (200 µM) or left untreated (normal) for 48 h. 5ʹtiRNA‐His‐GTG level was detected by qRT‐PCRs (*n* = 4; *p < 0.05, Student's *t*‐test). (G and H) mRNA and protein levels of ANG in Müller cells following exposure to H_2_O_2_ (200 µM) or control conditions (Ctrl) for 24 or 48 h, as detected by qRT‐PCRs (G; *n* = 4) and western blot (H; n = 3) (*p < 0.05, one‐way ANOVA with Bonferroni post hoc test). (I) Müller cells were transfected with ANG siRNA or scrambled control (Scr) for 24 h, and knockdown efficiency was detected by qRT‐PCRs (*n* = 4; *p < 0.05, Student's *t*‐test). (J) Müller cells transfected with ANG siRNA or Scr for 24 h, followed by quantification of 5ʹtiRNA‐His‐GTG levels using qRT‐PCRs (*n* = 4; *p < 0.05, Student's *t*‐test).

tsRNAs are classified based on cleavage sites into two major types: tiRNAs (tRNA halves) and tRFs, including 5ʹtRFs, 3ʹtRFs, and i‐tRFs (Figure 1A). Among these, 5ʹtiRNA‐His‐GTG is a 35‐nucleotide fragment derived from the 5′ end of mature tRNA‐His‐GTG (Figure 1B) and is highly conserved between mouse and human (Figure 1C).

To explore its potential involvement in retinal neurodegeneration, we examined 5ʹtiRNA‐His‐GTG expression in two established models of retinal neurodegeneration, including ONC and NMDA‐induced excitotoxicity. In both models, 5ʹtiRNA‐His‐GTG levels were significantly increased in both models (Figure 1D,E). Next, we compared 5ʹtiRNA‐His‐GTG expression across various retinal cell types, including RGCs, human retinal microvascular endothelial cells (HRMECs), Müller cells, pericytes, and retinal pigment epithelium (RPE). qRT‐PCR assays revealed that 5ʹtiRNA‐His‐GTG was predominantly expressed in Müller cells (Figure 1F).

Angiogenin (ANG), a stress‐responsive ribonuclease, is known to cleave the anticodon loop of mature tRNAs, generating tiRNAs [24]. To investigate whether ANG is involved in the production of 5ʹtiRNA‐His‐GTG, we examined its expression in Müller cells under oxidative stress. ANG mRNA and protein levels were significantly increased (Figure 1G,H). Silencing ANG significantly reduced ANG mRNA levels (Figure 1I) and led to decreased 5ʹtiRNA‐His‐GTG expression (Figure 1J), indicating that ANG is involved in the biogenesis of 5ʹtiRNA‐His‐GTG in Müller cells under stress conditions.

### 2.2 5ʹtiRNA‐His‐GTG Regulates Müller Cell Activity and Affects RGC Function In Vitro

**FIGURE 2:**
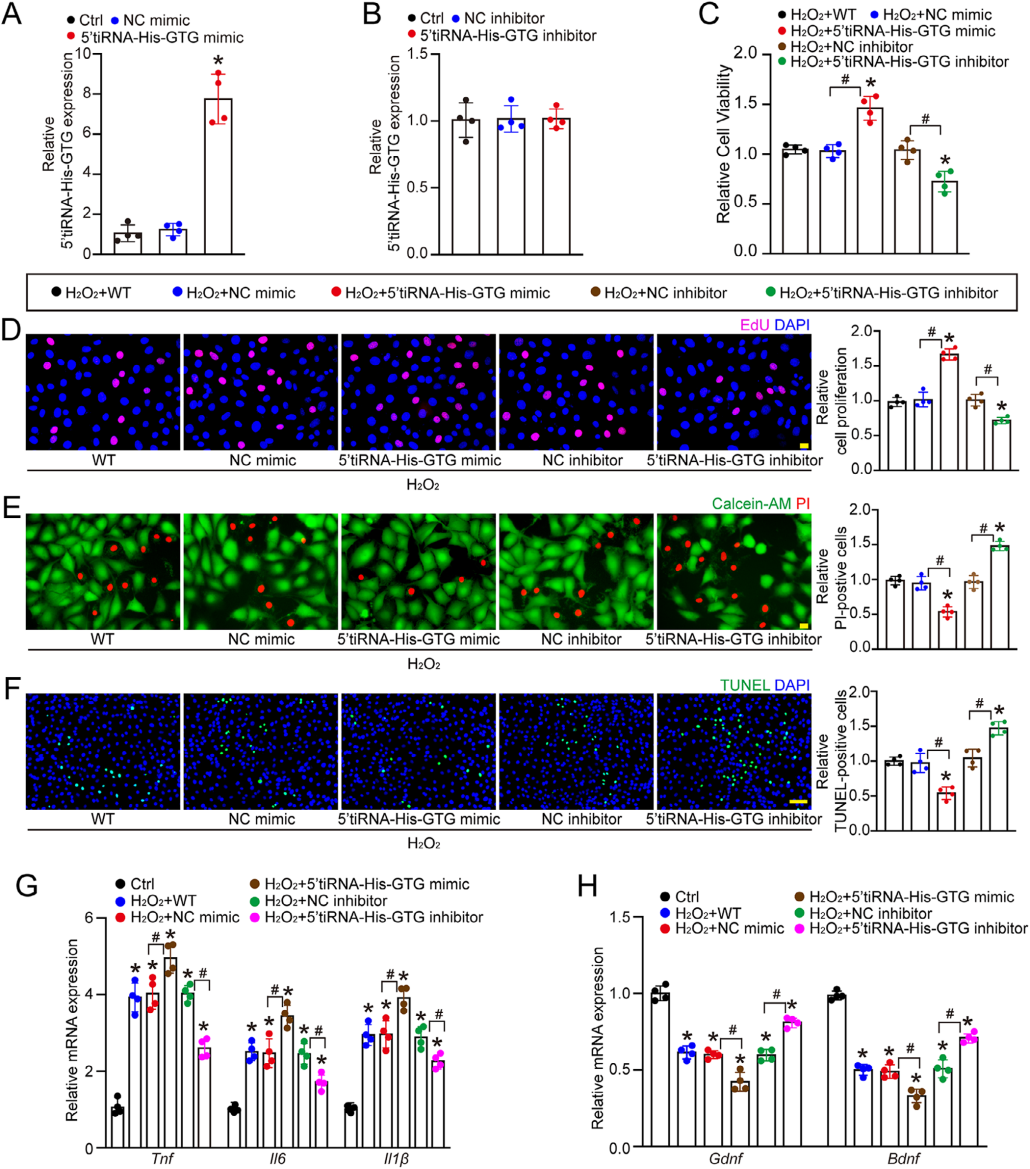
5ʹtiRNA‐His‐GTG regulates Müller cell activity and affects RGC function in vitro. (A and B) Müller cells were transfected with negative control (NC) mimic, 5ʹtiRNA‐His‐GTG mimic, NC inhibitor, or 5ʹtiRNA‐His‐GTG inhibitor, or left untreated (Ctrl) for 24 h. Expression levels of 5ʹtiRNA‐His‐GTG were detected by qRT‐PCRs (*n* = 4; *p < 0.05 versus NC mimic; one‐way ANOVA with Bonferroni post hoc test). (C–F) Müller cells were transfected with NC mimic, 5ʹtiRNA‐His‐GTG mimic, NC inhibitor, or 5ʹtiRNA‐His‐GTG inhibitor, or left untreated. After 6 h, the transfected Müller cells or left untreated (WT) were exposed to H_2_O_2_ (200 µM) for 48 h. The WT+H_2_O_2_ group served as the control for subsequent comparisons. (C) Cell viability was assessed by CCK‐8 assay (*n* = 4). (D) Cell proliferation was evaluated by EdU staining (EdU, red; DAPI, blue; scale bar: 20 µm; *n* = 4). (E) Cell death was assessed via Calcein‐AM/PI staining (Calcein‐AM, green; PI, red; scale bar: 20 µm; *n* = 4). (F) Apoptosis was analyzed by TUNEL assay (TUNEL, green; DAPI, blue; scale bar: 50 µm; *n* = 4). *p < 0.05 versus WT+H_2_O_2_; ^#^ *p* < 0.05 between indicated groups; one‐way ANOVA with Bonferroni post hoc test. (G and H) mRNA levels of *Tnf*, *Il6*, *Il1β*, *Gdnf*, and *Bdnf* were detected by qRT‐PCRs (*n* = 4). *p < 0.05 versus Ctrl; ^#^ *p* < 0.05 between indicated groups; one‐way ANOVA with Bonferroni post hoc test.

As the principal macroglia in the retina, Müller cells provide structural and metabolic support to retinal neurons, including photoreceptors and RGCs [10]. Primary Müller cells were isolated and confirmed by GS and GFAP staining, with >90% positivity (Figure S1A). Müller cells were transfected with 5ʹtiRNA‐His‐GTG mimics or inhibitors. qRT‐PCR confirmed successful modulation of 5ʹtiRNA‐His‐GTG levels. Overexpression increased intracellular 5ʹtiRNA‐His‐GTG, while inhibition had no effect on basal levels. LATS2 was predicted as a potential target based on sequence complementarity [25], and its expression was examined. Inhibition of 5ʹtiRNA‐His‐GTG increased LATS2 expression, suggesting a regulatory relationship between 5ʹtiRNA‐His‐GTG and LATS2 (Figure 2A,B; Figure S1B).

CCK‐8 assays revealed that inhibition of 5ʹtiRNA‐His‐GTG reduced Müller cell viability under oxidative stress, whereas overexpression increased cell viability (Figure 2C). EdU staining further demonstrated that cell proliferation decreased following 5ʹtiRNA‐His‐GTG inhibition and increased upon mimic treatment (Figure 2D). Calcein‐AM/PI staining and TUNEL assays demonstrated that 5ʹtiRNA‐His‐GTG inhibition exacerbated apoptosis, whereas mimic treatment conferred protection against H_2_O_2_‐induced cell death (Figure 2E,F).

Since reactive gliosis contributes to RGC damage by altering glial inflammatory and neurotrophic profiles [26, 27, 28], we next examined inflammatory and neurotrophic gene expression. Inhibition of 5ʹtiRNA‐His‐GTG decreased *Tnf*, *Il6*, and *Il1β*, while increasing *Bdnf* and *Gdnf*. Opposite changes were observed after overexpression (Figure 2G,H). To assess Müller–RGC interaction, primary RGCs were co‐cultured with Müller cells and identified by Tuj1 staining (>90% positive; Figure S2A). Under oxidative stress, Müller cells reduced RGC viability and increased PI‐positive cells. These effects were enhanced by 5ʹtiRNA‐His‐GTG overexpression and attenuated by inhibition (Figure S2B–D).

Overall, 5ʹtiRNA‐His‐GTG regulates Müller cell activation and inflammatory status, and indirectly influences RGC survival under stress conditions.

### 2.3 5ʹtiRNA‐His‐GTG Regulates Retinal Neurodegeneration In Vivo

**FIGURE 3:**
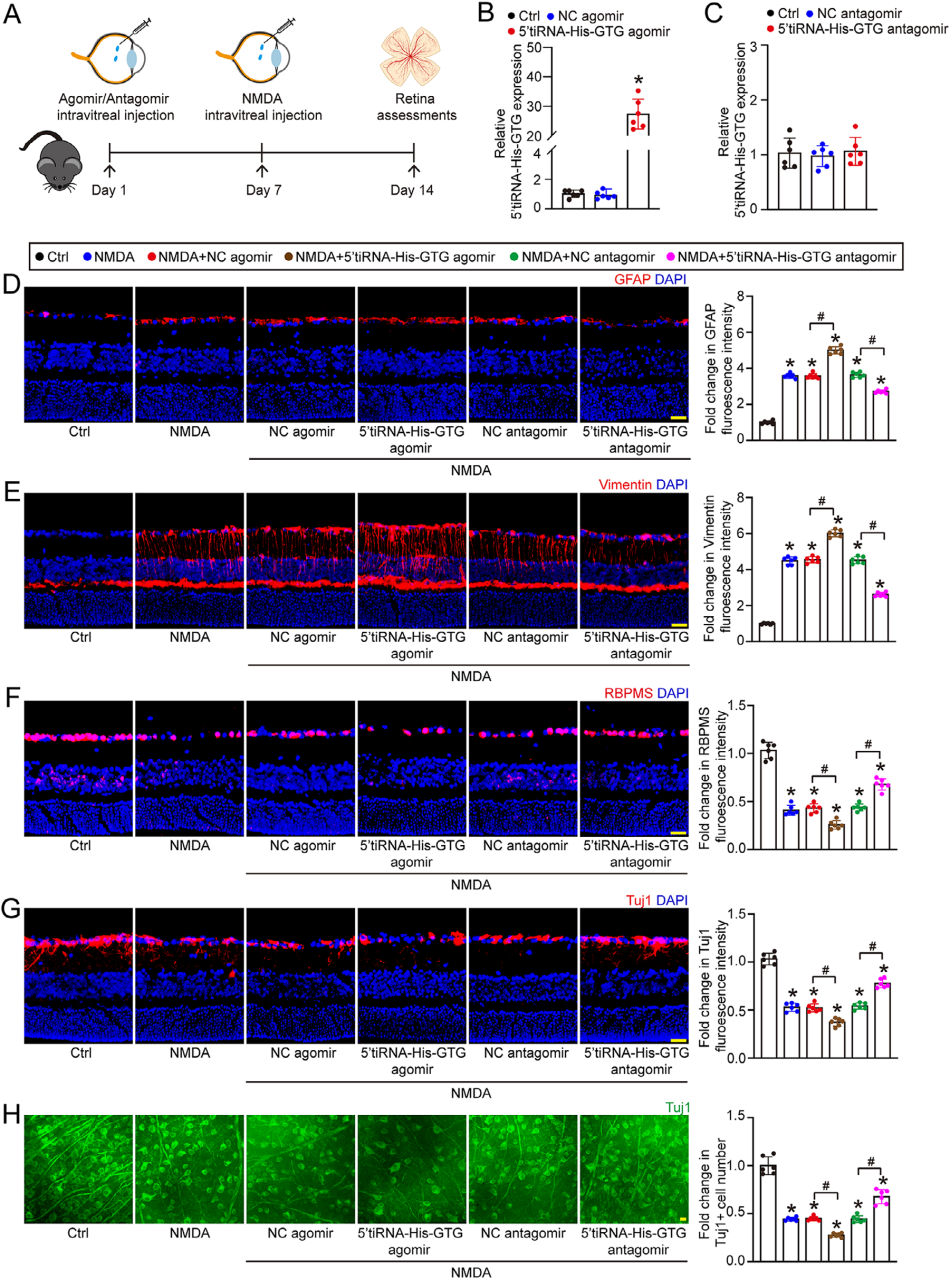
5ʹtiRNA‐His‐GTG regulates retinal neurodegeneration in vivo. (A) Schematic illustration of the experimental workflow for panels B–H. (B and C) Eight‐week‐old C57BL/6 male mice received intravitreal injections of negative control (NC) agomir, 5ʹtiRNA‐His‐GTG agomir, NC antagomir, or 5ʹtiRNA‐His‐GTG antagomir, while untreated mice served as controls (Ctrl). One week after injection, retinal tissues were collected, and 5ʹtiRNA‐His‐GTG levels were detected by qRT‐PCRs (*n* = 6; *p < 0.05 versus NC agomir; one‐way ANOVA with Bonferroni post hoc test). (D and E) One week after oligonucleotide injection, NMDA (20 mM, 1.5 µL) was intravitreally administered. Retinas were harvested one week later for immunofluorescence staining of GFAP (D) and vimentin (E) to assess reactive gliosis (*n* = 6). Nuclei were stained with DAPI (blue). Scale bars, 50 µm. (F and G) Immunofluorescence staining for RBPMS (F) and Tuj1 (G) was performed to detect RGC survival (*n* = 6). Nuclei were stained with DAPI (blue). Scale bars, 50 µm. (H) Retinal whole‐mount staining with Tuj1 was used to visualize and quantify surviving RGCs (*n* = 6). Scale bar, 20 µm. *p < 0.05 versus Ctrl; ^#^ *p* < 0.05 between indicated groups. Statistical analysis was performed using one‐way ANOVA with Bonferroni post hoc test.

We used two mouse models of retinal neurodegeneration, NMDA‐induced excitotoxicity and ONC, to examine the role of 5ʹtiRNA‐His‐GTG. NMDA injection induces glutamate‐mediated retinal injury, while ONC causes acute axonal damage. 5ʹtiRNA‐His‐GTG agomir or antagomir was delivered intravitreally to modulate its expression. qRT‐PCR confirmed effective regulation of 5ʹtiRNA‐His‐GTG levels (Figure 3A–C; Figure S3A).

Retinal neurodegeneration is characterized by reactive gliosis and RGC loss [29, 30]. In the NMDA model, immunofluorescence staining showed that 5ʹtiRNA‐His‐GTG agomir increased GFAP and vimentin expression, indicating enhanced glial activation, whereas antagomir reduced this response (Figure 3D,E). In parallel, agomir treatment reduced the number of Tuj1^+^ and RBPMS^+^ RGCs, while antagomir preserved RGC survival (Figure 3F,G). Retinal whole‐mount analysis further confirmed that inhibition of 5ʹtiRNA‐His‐GTG improved RGC preservation (Figure 3H). Similar protective effects of 5ʹtiRNA‐His‐GTG inhibition were observed in the ONC model, further supporting its role across distinct types of retinal neurodegeneration (Figure S3B–G).

Taken together, these results suggest that inhibition of 5ʹtiRNA‐His‐GTG reduced reactive gliosis and preserved RGC survival in both excitotoxic and traumatic injury models, suggesting its potential as a therapeutic target in retinal neurodegeneration.

### 2.4 5ʹtiRNA‐His‐GTG Regulates Visual Function In Vivo

**FIGURE 4:**
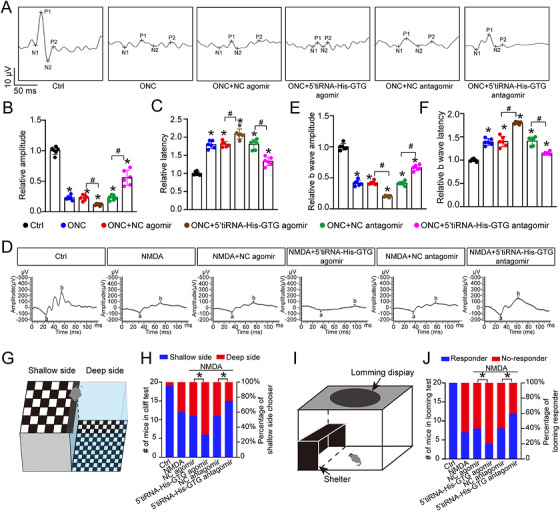
5ʹtiRNA‐His‐GTG regulates visual function in vivo. (A) Representative flash visual evoked potential (FVEP) waveforms from control (Ctrl) mice, optic nerve crush (ONC) mice, and ONC mice receiving intravitreal injections of negative control (NC) agomir, 5ʹtiRNA‐His‐GTG agomir, NC antagomir, or 5ʹtiRNA‐His‐GTG antagomir. (B and C) Quantification of N1‐P1 amplitude (B) and P1 latency (C) from FVEP recordings (*n* = 6). (D) Representative flash electroretinography (ERG) waveforms from control (Ctrl) mice, NMDA‐treated mice, and NMDA‐treated mice receiving intravitreal injections of NC agomir, 5ʹtiRNA‐His‐GTG agomir, NC antagomir, or 5ʹtiRNA‐His‐GTG antagomir. (E and F) Quantification of b‐wave amplitude (E) and latency (F) from ERG recordings (*n* = 6). (G) Schematic illustration of the visual cliff assay for assessing depth perception. (H) Behavioral outcomes of the visual cliff assay, presented as both the number (left *y*‐axis) and percentage (right *y*‐axis) of mice selecting the cliff or safe side (*n* = 20). (I) Schematic illustration of looming visual stimulus paradigm used to assess innate visual responses. (J) Quantification of looming responses, presented as the number (left *y*‐axis) and percentage (right *y*‐axis) of mice exhibiting escape or freezing behaviors (*n* = 20). *p < 0.05 versus Ctrl; ^#^ *p* < 0.05 between indicated groups. Statistical analysis was performed using one‐way ANOVA with Bonferroni post hoc test.

Visual evoked potential (VEP) was used to assess signal transmission from RGCs to the visual cortex. Flash VEP (FVEP) recordings were performed in the ONC model. Two weeks after injury, ONC mice showed reduced N1–P1 amplitudes and prolonged P1 latency. These deficits were aggravated by 5ʹtiRNA‐His‐GTG agomir treatment, whereas antagomir partially restored FVEP responses, with increased N1–P1 amplitude and reduced P1 latency (Figure 4A–C). Electroretinography (ERG) was performed to assess retinal function. In the NMDA‐induced excitotoxicity model, b‐wave amplitude was reduced and latency was prolonged, indicating impaired inner retinal activity. 5ʹtiRNA‐His‐GTG agomir further aggravated these changes, whereas antagomir partially restored b‐wave amplitude and latency (Figure 4D–F).

To assess visual behavior, depth perception was evaluated using a visual cliff assay. NMDA‐treated mice showed impaired depth discrimination, which was further exacerbated by 5′tiRNA‐His‐GTG agomir administration. In contrast, antagomir treatment improved performance (Figure 4G,H). Innate visual responses were examined using a looming visual stimulus test. Under physiological conditions, mice typically respond to looming stimuli with escape or freezing behaviors. However, 5′tiRNA‐His‐GTG agomir significantly attenuated these defensive responses, whereas antagomir treatment partially rescued them (Figure 4I,J).

Collectively, these results indicate that inhibition of 5ʹtiRNA‐His‐GTG preserves retinal visual function under excitotoxic and traumatic injury conditions.

### 2.5 5ʹtiRNA‐His‐GTG Regulates Retinal Neurodegeneration via Glycerophospholipid Metabolism

**FIGURE 5:**
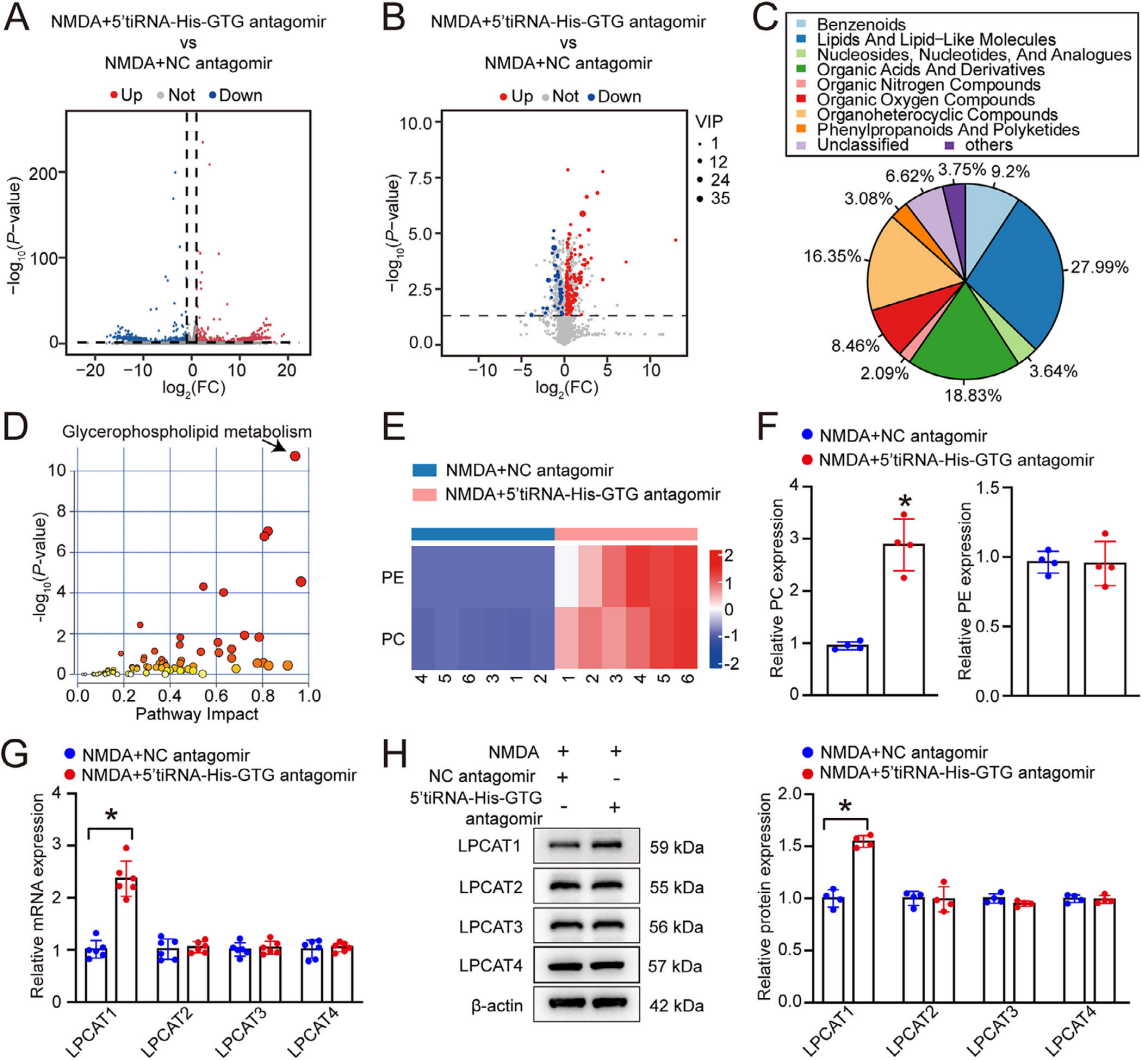
5ʹtiRNA‐His‐GTG regulates retinal neurodegeneration via glycerophospholipid metabolism. (A–C) Eight‐week‐old male C57BL/6 mice received intravitreal injections of 5ʹtiRNA‐His‐GTG antagomir or negative control (NC) antagomir. One week later, NMDA (20 mM, 1.5 µL per eye) was administered via intravitreal injection. Retinas were collected one week after NMDA treatment. Volcano plot showing differentially expressed genes between groups (*n* = 3). Significance thresholds were defined as |log2 fold change (FC)| > 1 and *p* < 0.05. Upregulated genes are shown in red and downregulated genes in blue (A). Volcano plot showing differentially expressed metabolites between groups (*n* = 6). Selection criteria were variable importance in projection (VIP) > 1, |log2(FC)| > 1, and *p* < 0.05. Upregulated metabolites are shown in red and downregulated metabolites in blue (B). Pie chart summarizing chemical classification of differential metabolites, with lipids and lipid‐like molecules representing the predominant category (C). (D) Integrated pathway enrichment analysis of significantly altered transcripts and metabolites. The *x*‐axis represents pathway impact scores derived from topology analysis, while the *y*‐axis and bubble color indicate statistical significance. Glycerophospholipid metabolism was highlighted as one of the most significantly altered pathways. (E) Heatmap showing relative abundance of phosphatidylethanolamine (PE) and phosphatidylcholine (PC). Red indicates higher levels and blue indicates lower levels. (F) Quantification of PC and PE levels in mouse retinas by ELISA assays (*n* = 4). (G and H) Expression levels of LPCAT1‐4 were detected by qRT‐PCRs (G, *n* = 4) and western blot (H, *n* = 4), *p < 0.05. Statistical analysis was performed using one‐way ANOVA with Bonferroni post hoc test.

NMDA‐induced excitotoxicity was used as a model of retinal neurodegeneration. Mice were intravitreally injected with 5ʹtiRNA‐His‐GTG antagomir or control antagomir before NMDA treatment. Retinas were harvested for transcriptomic and metabolomic analyses. Principal component analysis (PCA) and orthogonal partial least squares discriminant analysis (OPLS‐DA) showed clear separation between groups, indicating substantial transcriptomic (Figure S4A) and metabolic (Figure S4B) alterations upon 5ʹtiRNA‐His‐GTG inhibition. RNA sequencing identified 559 upregulated and 552 downregulated genes (Figure 5A), while untargeted metabolomics detected 127 increased and 48 decreased metabolites (Figure 5B). Lipids and lipid‐like molecules represented the predominant class of altered metabolites (Figure 5C). Integrated analysis of transcriptomic and metabolomic data revealed significant alterations in glycerophospholipid metabolism during NMDA‐induced retinal injury (Figure 5D), suggesting a key role in 5ʹtiRNA‐His‐GTG‐mediated effects.

To further explore whether alterations in glycerophospholipid metabolism may also occur in other neurodegenerative diseases, we analyzed single‐cell transcriptomic data from Alzheimer's disease (AD) brain samples. This analysis revealed a similar enrichment of glycerophospholipid metabolism in macroglial populations (Figure S4C,D), supporting the notion that dysregulation of this pathway may represent a shared metabolic signature across neurodegenerative conditions. Metabolomic analysis identified phosphatidylethanolamine (PE) and phosphatidylcholine (PC) as key altered metabolites within the glycerophospholipid pathway (Figure 5E). Enzyme‐Linked Immunosorbent Assay (ELISA)‐based quantification showed increased PC levels following 5ʹtiRNA‐His‐GTG antagomir treatment, whereas PE levels remained unchanged (Figure 5F).

In addition, Mendelian randomization (MR) analysis revealed a significant association between PC‐associated SNPs and glaucoma risk (Figure S4E). These associations were consistent across heterogeneity and pleiotropy analyses (Figure S4F) and were supported by leave‐one‐out validation (Figure S4G), suggesting a potential protective association of PC in neurodegenerative processes.

To further investigate the molecular basis of PC regulation, we examined lysophosphatidylcholine acyltransferases (LPCATs), key enzymes involved in PC biosynthesis. Among the four isoforms (LPCAT1‐4), transcriptomic analysis revealed selective upregulation of LPCAT1 following 5ʹtiRNA‐His‐GTG inhibition, which was validated by qRT‐PCR and western blot (Figure 5G,H).

### 2.6 5ʹtiRNA‐His‐GTG Modulates Retinal Neurodegeneration Through the LPCAT1‐PC Pathway

**FIGURE 6:**
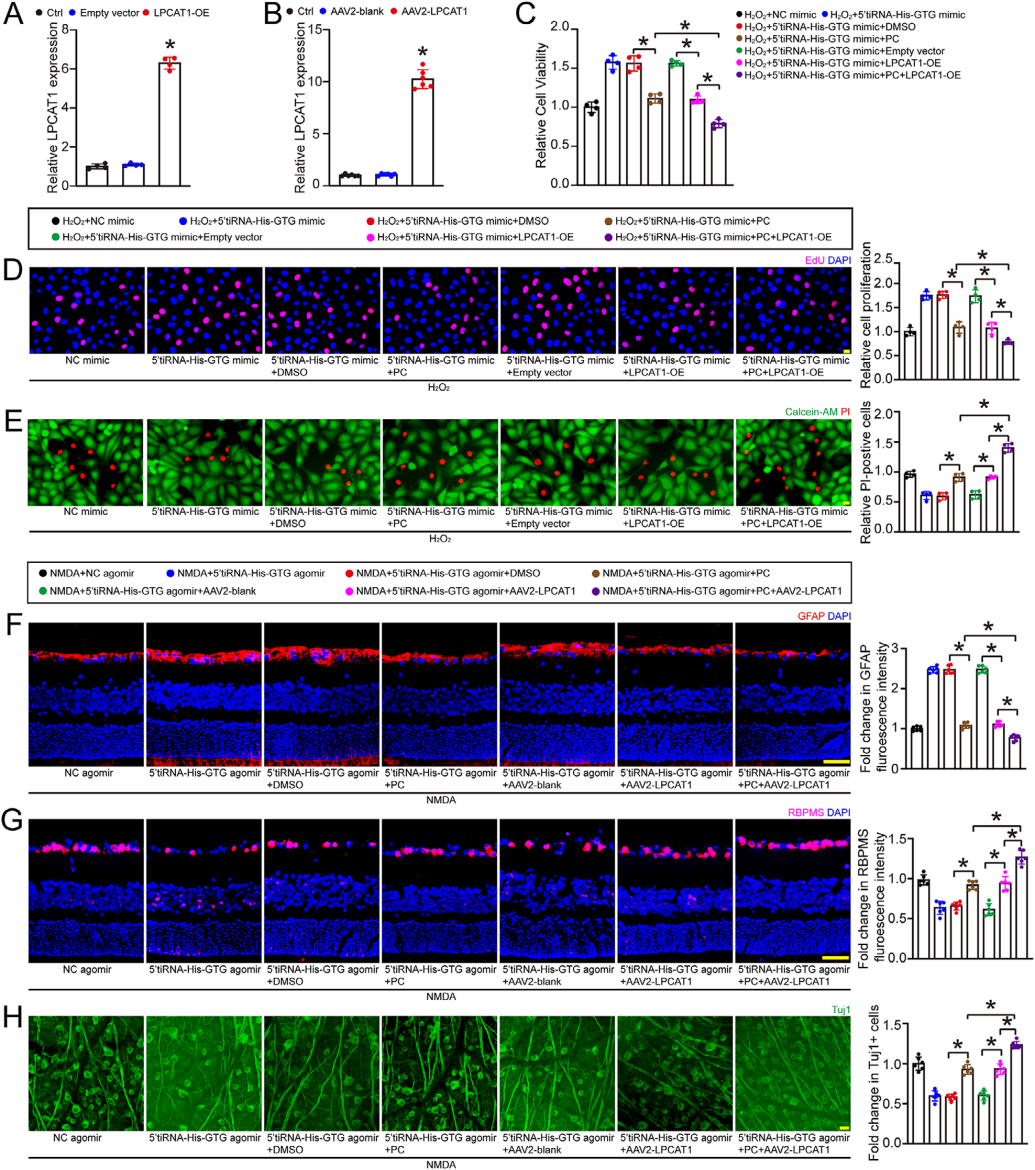
5ʹtiRNA‐His‐GTG modulates retinal neurodegeneration through the LPCAT1‐PC pathway. (A) qRT‐PCR analysis of LPCAT1 mRNA levels in Müller cells transfected with empty vector, LPCAT1‐overexpression vector (LPCAT1‐OE), or left untreated (Ctrl) (*n* = 4; *p < 0.05 vs. empty vector; one‐way ANOVA with Bonferroni post hoc test). (B) qRT‐PCR analysis of LPCAT1 mRNA levels in mouse retinas following intravitreal injection of AAV2‐blank or AAV2‐LPCAT1 (*n* = 6; *p < 0.05 vs. AAV2‐blank). (C–E) Müller cells were transfected with negative control (NC) mimic, 5ʹtiRNA‐His‐GTG mimic, or 5ʹtiRNA‐His‐GTG mimic combined with phosphatidylcholine (PC, 50 µM), DMSO, empty vector, or LPCAT1‐OE, followed by exposure to H_2_O_2_ for 48 h. H_2_O_2_ + NC mimic group served as the control for comparisons. Cell viability was assessed by CCK‐8 assay (C, *n* = 4). Cell proliferation was evaluated by EdU incorporation assay. EdU, red; DAPI, blue. Scale bar, 20 µm (D, *n* = 4). Cell death was assessed by Calcein‐AM/PI double staining. Calcein‐AM, green; PI, red. Scale bar, 20 µm (E, *n* = 4). (F–H) Eight‐week‐old male C57BL/6 mice received intravitreal injections of NC agomir, 5ʹtiRNA‐His‐GTG agomir, or 5ʹtiRNA‐His‐GTG agomir combined with DMSO, PC (100 µM), or AAV2‐LPCAT1, followed by NMDA (20 mM, 1.5 µL per eye) administration. The NMDA + NC agomir group served as the control for comparisons. (F) Immunofluorescence staining and quantification of GFAP expression to assess reactive gliosis. DAPI, blue. Scale bar, 50 µm (F, *n* = 6). (G) Immunofluorescence staining and quantification of RBPMS‐positive RGCs. DAPI, blue. Scale bar, 50 µm (G, *n* = 6). (H) Whole‐mount retinal staining and quantification of Tuj1‐positive RGCs. Scale bar, 20 µm (H, *n* = 6). *p < 0.05 between indicated groups. Statistical analysis was performed using one‐way ANOVA with Bonferroni post hoc test.

To determine whether LPCAT1‐mediated PC metabolism contributes to the biological effects of 5ʹtiRNA‐His‐GTG, LPCAT1 overexpression was established in vitro and in vivo. In Müller cells, plasmid‐mediated LPCAT1 overexpression significantly increased LPCAT1 mRNA levels (Figure 6A). AAV‐mediated delivery similarly elevated LPCAT1 expression in mouse retinas (Figure 6B).

We next examined whether modulation of LPCAT1‐PC axis affects Müller cell responses to 5ʹtiRNA‐His‐GTG. 5ʹtiRNA‐His‐GTG overexpression increased Müller cell viability and proliferation, as shown by CCK‐8 and EdU assays. These effects were reduced by LPCAT1 overexpression or PC supplementation. Combined treatment showed a stronger effect on cell proliferation (Figure 6C,D). Under oxidative stress, LPCAT1 overexpression increased cell death in Müller cells, which was further enhanced by PC supplementation (Figure 6E), indicating that LPCAT1‐PC modulation alters stress responses.

In the Müller cell‐RGC co‐culture system, 5ʹtiRNA‐His‐GTG‐overexpressing Müller cells increased RGC death under H_2_O_2_ treatment. This effect was attenuated by either LPCAT1 overexpression or PC supplementation in Müller cells (Figure S5), suggesting that LPCAT1‐PC metabolism in Müller cells influences RGC survival indirectly. In vivo, both LPCAT1 overexpression and PC supplementation reduced GFAP‐positive gliosis (Figure 6F) and preserved RGCs, as shown by increased RBPMS^+^ cells (Figure 6G). Whole‐mount staining confirmed these effects (Figure 6H). Combined treatment showed a more pronounced effect on gliosis and RGC preservation.

Overall, these data indicate that 5ʹtiRNA‐His‐GTG regulates Müller cell activity and RGC survival through the LPCAT1‐PC pathway. Modulation of this axis contributes to neuroprotective effects observed upon 5ʹtiRNA‐His‐GTG inhibition.

## 3 Discussion

Retinal neurodegenerative diseases, such as glaucoma, traumatic optic neuropathy, and diabetic retinopathy, represent major causes of irreversible visual impairment worldwide and impose a substantial public health burden [31]. These diseases are generally characterized by a progressive loss of retinal neurons, especially RGCs, along with chronic inflammation, dysfunction of glial cells, and metabolic disturbance. Although current clinical methods, such as lowering intraocular pressure or improving metabolic control, can slow disease progression, they cannot effectively stop or reverse neuronal loss. Thus, there is a pressing need to identify new molecular regulators and potential therapeutic targets to enable effective neuroprotective strategies.

tsRNAs have emerged as important epigenetic regulators involved in diverse biological processes, including translational regulation, stress responses, and apoptosis [32, 33]. Among these, tiRNAs are generated through ANG‐mediated cleavage at the anticodon loop of mature tRNAs, producing fragments of 30–35 nucleotides [34, 35]. Accumulating evidence indicates that aberrant tiRNA expression is implicated in the pathogenesis of cancers, metabolic diseases, and neurodegenerative diseases such as ALS and AD [36, 37, 38, 39]. Notably, tiRNAs often exhibit tissue‐ and stress‐specific expression patterns and modulate cellular survival under oxidative, inflammatory, or metabolic stress. In ocular systems, altered tiRNA profiles have been reported in models of diabetic retinopathy and age‐related macular degeneration [38, 40, 41]. In this study, we found that 5′tiRNA‐His‐GTG was consistently upregulated in both NMDA‐induced excitotoxic and ONC models of retinal neurodegeneration, as well as in Müller cells exposed to oxidative stress, suggesting a robust and stress‐responsive regulatory role.

RGCs are the primary output neurons of the retina, integrating visual signals from photoreceptors and transmitting them to central visual centers via optic nerve. Because mature RGCs exhibit limited regenerative capacity, their loss is central to the pathophysiology of most retinal neurodegenerative diseases [42]. In our study, 5′tiRNA‐His‐GTG was found to contribute to RGC degeneration. Notably, inhibition of 5′tiRNA‐His‐GTG preserved RGC survival and improved visual function, underscoring its functional importance and therapeutic potential.

Reactive gliosis is another hallmark of retinal injury. Under physiological conditions, Müller cells provide structural support, maintain ionic balance, and supply metabolic substrates to neurons. However, in response to injury, they undergo morphological and molecular reprogramming characterized by upregulation of GFAP and vimentin, secretion of pro‐inflammatory cytokines, and impaired neurotrophic support [43, 44, 45]. We show that 5′tiRNA‐His‐GTG is markedly upregulated in Müller cells under oxidative stress. Its overexpression promotes glial proliferation, enhances inflammatory activation, and strengthens anti‐apoptotic signaling, whereas inhibition of 5′tiRNA‐His‐GTG suppresses gliosis and helps restore Müller cell homeostasis. In addition, co‐culture experiments further demonstrated that 5′tiRNA‐His‐GTG modulates Müller cell–RGC interactions, thereby exacerbating or alleviating RGC injury depending on its expression level.

To investigate the underlying mechanism of 5′tiRNA‐His‐GTG, we performed integrated metabolomic and transcriptomic analyses. Notably, glycerophospholipid metabolism emerged as one of the most significantly affected pathways, with a particular increase in PC levels and upregulation of its biosynthetic enzyme LPCAT1. Glycerophospholipids are essential components of cellular membranes and play key roles in membrane dynamics, signal transduction, and the regulation of apoptosis [46, 47]. Phosphatidylcholine serves as a precursor for bioactive lipid mediators and is essential for maintaining mitochondrial integrity and supporting neuronal survival. Importantly, either exogenous PC supplementation or LPCAT1 overexpression reversed the pathological effects induced by 5′tiRNA‐His‐GTG, restoring Müller cell viability, suppressing glial activation, and promoting RGC survival. These results suggest that 5′tiRNA‐His‐GTG contributes to retinal neurodegeneration, at least in part, through suppression of LPCAT1‐PC axis.

MR analysis based on large‐scale public genomic datasets revealed a significant inverse association between single nucleotide polymorphisms involved in phosphatidylcholine metabolism and the risk of glaucoma. This genetic evidence is consistent with our mechanistic findings and suggests that enhanced phosphatidylcholine biosynthesis may exert a protective effect on retinal integrity by limiting neurodegenerative processes. Collectively, these results further support the importance of the LPCAT1‐mediated PC synthesis pathway in maintaining RGC survival. Given that the retina is an anatomical and developmental extension of the central nervous system, we next explored whether similar metabolic alterations might be present in other neurodegenerative conditions. In an exploratory analysis of published single‐cell RNA‐seq datasets from AD brains, we observed a comparable downregulation of glycerophospholipid metabolism in astrocytes, partially mirroring metabolic changes identified in retinal Müller glia. Importantly, these observations are associative and derived from independent datasets. While our study primarily focuses on retinal neurodegeneration, this cross‐dataset comparison suggests that disrupted glial lipid metabolism may represent a broader feature of CNS neurodegenerative disorders, warranting further investigation.

Although our findings suggest that targeting 5′tiRNA‐His‐GTG holds therapeutic potential for preserving vision, several issues need to be considered before clinical application. RNA‐based molecules are prone to degradation by nucleases in vivo. In practice, chemical modifications such as 2’‐O‐methylation and phosphorothioate backbones are commonly used to improve molecular stability and specificity [48]. Another important challenge is achieving efficient and cell‐specific delivery to Müller glial cells. The engineered adeno‐associated virus ShH10 variant, which shows preferential tropism for Müller cells, may provide a feasible approach for targeted delivery [49]. In addition, given the sequence similarity between tRNA‐derived fragments and their parental tRNAs, there is a potential risk of unintended interactions with non‐target RNAs. Thus, comprehensive transcriptome‐wide assessment of off‐target effects, along with rigorous preclinical safety evaluation, will be required prior to clinical translation.

Collectively, our study identifies 5′tiRNA‐His‐GTG as a previously unrecognized stress‐responsive non‐coding RNA that contributes to retinal neurodegeneration through glial activation and metabolic reprogramming. Its upregulation is associated with enhanced gliosis, reduced phosphatidylcholine synthesis, and increased RGC loss, whereas its inhibition promotes neuronal survival and preserves visual function. These findings provide new insight into the pathogenesis of retinal degeneration and highlight 5′tiRNA‐His‐GTG as a promising therapeutic target in neurodegenerative diseases. Future studies should further assess its therapeutic potential in human retinal organoids and explore gene therapy‐based strategies for clinical translation.

## 4 Methods and Materials

### 4.1 Mice Manipulation

For the ONC model, 8‐week‐old male mice were anesthetized and treated with a drop of 0.5% proparacaine hydrochloride. Conjunctival peritomy was performed at the limbus and the optic nerve was crushed with forceps for 10 s at approximately 1 mm posterior to the globe. After the procedure, the mice were treated with antibiotics to prevent infection. The NMDA‐induced injury model was established using NMDA (20 mM; 454575, Millipore Sigma, Canada). Mice were anesthetized, and the pupils were dilated by 1% tropicamide (Akorn Pharmaceuticals, Illinois, USA). A volume of 1.5 µL of NMDA was injected into the vitreous cavity using a microinjector fitted with a 33‐gauge needle (Hamilton, Switzerland). Following injection, mice received antibiotic treatment. Animals that did not survive the procedure were excluded from analysis.

### 4.2 Cell Isolation and Culture

Primary Müller glial cells were isolated from postnatal Day 7–10 mice. Briefly, mice were euthanized, and eyes were enucleated under sterile conditions. Retinas were carefully dissected and digested in 0.25% trypsin (T9201, Sigma, USA) at 37°C for 30 min with gentle trituration. Digestion was terminated by adding an equal volume of DMEM‐F12 containing 12% fetal bovine serum (FBS; FBS500‐S, AusGeneX, Australia). Following this, the cell suspension was filtered through a 30‐µm strainer, centrifuged at 300 × *g* for 5 min, and resuspended in DMEM/F12 medium containing 12% fetal bovine serum before plating onto poly‐l‐lysine–coated dishes. Non‐adherent cells were removed after 24 h, and the medium was replaced every 2–3 days. Müller cells were enriched through differential adhesion and repeated passaging. The purity of Müller cells was confirmed by immunostaining for GS and GFAP.

Primary RGCs were extracted from postnatal Day 3 mice. The retinas were treated with a papain solution (5 mg/mL) for 30 min at 37°C. Subsequently, the cells underwent purification through negative selection using anti‐macrophage antibody (CLAD31240, Cedarlane, Canada) and positive selection using anti‐Thy1.2 antibody (MCA02R, Bio‐Rad, USA). The purified RGCs were then suspended in complete medium (CM‐M122, Procell, China) and seeded onto 24‐well plates precoated with poly‐d‐lysine to enhance cell adhesion. Cells were maintained at 37°C in a humidified incubator with 5% CO_2_.

### 4.3 Immunochemistry

Following enucleation, eyeballs were fixed in Fekete's solution for 2 h at room temperature, followed by fixation in 4% paraformaldehyde at 4°C for 24 h. Retinal tissues were cryoprotected in 30% sucrose for 48 h and embedded in OCT. Sections (10 µm) were prepared using a cryostat. Sections were blocked with 5% BSA and 1% Triton X‐100 for 45 min at 37°C. Sections were incubated with primary antibodies (Table S1) overnight at 4°C. Slides were washed twice with PBST and incubated with Alexa Fluor 594 goat anti‐rabbit IgG (A11012, Thermo Fisher Scientific, USA) for 2 h. DAPI was used for nuclear staining. Fluorescence was imaged using a fluorescence microscope and quantified with ImageJ (v1.53c).

### 4.4 Electrophysiology

For ONC models, retinal functional assessments were conducted 14 days post‐modeling. For NMDA models, retinal functional assessments were conducted 7 days post‐modeling. Following overnight dark adaptation, animals were anesthetized via intraperitoneal injection of ketamine/xylazine (80/10 mg/kg). Pupils were dilated with 1% tropicamide, and corneal anesthesia was achieved with 0.5% proparacaine hydrochloride. F‐VEP recordings were performed using subdermal needle electrodes positioned at the interaural midpoint. ERG recordings were performed using corneal loop electrodes with reference electrodes placed in the cheek. A tail electrode served as ground. White light flashes were delivered at 3 cd·s/m^2^ with a 5 ms interstimulus interval. The contralateral eye was covered during recording. Retinal responses were recorded using the GOTEC visual testing system (GOTEC Medical, Chongqing, China). Amplitudes and latencies were calculated automatically. For electrophysiological recordings, at least six mice were included per group. To ensure objectivity and minimize bias, the experiments were performed in a blinded manner. Random numbers were generated using an online random order generator (https://www.graphpad.com/quickcalcs/randomize1/). The investigator performing the ERG/F‐VEP recordings and the subsequent waveform analyses (measuring b‐wave amplitudes or P1 latencies) was masked to the treatment conditions. Animal groups were coded with random numbers, and the code was broken only after the data analysis was completed.

### 4.5 Visual Cliff Test

The visual cliff apparatus consisted of a transparent platform dividing a shallow and deep side, creating a depth illusion. Mice were placed on the central platform, and their choice was recorded. Each mouse was tested once, and the apparatus was cleaned between trials.

### 4.6 Looming Visual Stimuli Response Test

The looming visual stimulus response test was performed in a plexiglass enclosure equipped with a shelter area for escape behavior. A display screen was positioned above the enclosure to present looming stimuli. The visual stimulus consisted of an expanding black circle (5–40 cm) over 0.25 s, followed by a 0.25 s plateau. This stimulus was repeated 15 times at 0.5 s intervals. Mice were acclimated to the enclosure for 5 min before testing.

### 4.7 Enzyme‐Linked Immunosorbent Assay

ELISA kits (CB11816‐Mu and CB14932‐Mu, COIBO BIO, China) were used to quantify PC or PE. Retinal supernatant was diluted 1:5 with the provided diluent and added to ELISA plates. Plates were incubated with detection antibody for 1 h. After incubation, wells were washed five times with wash buffer to remove unbound antibodies. Subsequently, 100 µL of substrate mixture was added to each well. This substrate mixture reacts with the enzyme linked to the detection antibody to produce a colorimetric change that is proportional to the concentration of phosphatidylcholine in the sample. The plate was incubated for an additional 15 min, and the reaction was stopped by adding stop solution. Optical density was measured at 450 nm using a FilterMax F5 microplate reader.

### 4.8 Dataset Acquisition

This study used a publicly available single‐cell RNA sequencing (scRNA‐seq) dataset related to AD obtained from the Gene Expression Omnibus (GEO) database: GSE175814. GSE175814 contains single‐cell transcriptomic data from two AD samples (Braak stage 3, brain regions: BA41/42, BA 6/8, and anterior hippocampal cortex) and two control samples. scRNA‐seq data were analyzed using the Seurat package (version 3.2.2) in R (version 4.0.2). The analysis included quality control, dimensionality reduction, and visualization.

### 4.9 Single‐Cell Data Processing

Data integration was performed using the Harmony package to correct batch effects. The Read10X function in Seurat was used to load sample data and create an aggregated Seurat object. After normalization, highly variable genes were selected using an established statistical approach considering dispersion and mean–variance relationships. Subsequently, scaling and PCA were performed using the top 1000 most variable genes.

Seurat‐based quality control was applied, including filtering cells with < 350 or > 3000 detected genes, and excluding cells with > 20% mitochondrial gene content.

To identify cluster‐specific markers, the FindAllMarkers function was used. Additionally, major cell type markers (≥ 1.5‐fold change) were identified based on CellMarker databases or previous studies.

### 4.10 Molecular Network Analysis of Differential Metabolites and Genes

Differential gene expression analysis was performed using the limma package (in a pseudo‐bulk framework). Integrated network analysis of metabolites and genes was performed using MetaboAnalyst 5.0 to identify associated metabolic and signaling pathways.

### 4.11 MR Analysis

MR analysis was used to assess causal relationships between genetic factors (as instrumental variables) and phenotypic outcomes, thereby reducing confounding and reverse causation bias in observational studies. The primary analysis used the inverse variance weighted (IVW) method, which combines estimates from multiple genetic instruments to provide an overall causal effect. To assess the robustness of the results, two additional MR methods were applied: the weighted median approach, which provides reliable estimates even when some instruments are invalid, and MR‐Egger regression, which accounts for potential pleiotropy by incorporating an intercept term.

## Author Contributions

Biao Yan designed the study. Yuke Ji, Sha Liu, Ying Zhang, Junya Zhu, Chang Huang, Ya Zhao, Jiao Xia, and Wan Mu conducted the experiments and acquired data. Yuke Ji and Sha Liu analyzed the results and prepared figures. Yuke Ji, Junya Zhu, and Biao Yan wrote the manuscript. Yuke Ji, Sha Liu, Ya Zhao, and Junya Zhu developed the methods and data analysis. Biao Yan, Jin Yao, and Wan Mu contributed to the critical discussion of results. All authors have read and approved the final manuscript.

## Funding

This study was supported by the grants from the National Natural Science Foundation of China (nos.81770945 and 81970809 to Biao Yan), the Fundamental Research Funds for the Central Universities (YG2025ZD04 to Biao Yan), and the Shanghai Municipal Health Commission Collaborative Innovation Cluster Project (2024CXJQ02 to Biao Yan).

## Ethics Statement

All procedures and protocols were approved by the Animal Ethics Committee of Nanjing Medical University (Approval Number: IACUC‐2407049).

## Conflicts of Interest

The authors declare no conflicts of interest.

## Supporting information



Supporting file 1: mco270879‐sup‐0001‐SuppMat.pdf

## Data Availability

The data sets generated and analyzed during this study are available from the corresponding author upon reasonable request.
