# Supplementary material for: Reprogramming Glial Cell Metabolism via a tRNA Fragment Preserves Vision in Retinal Neurodegeneration
Source: MedComm (2020). 2026 Jul 22;7(8):e70879. doi: 10.1002/mco2.70879 (PMC13392496; doi:10.1002/mco2.70879)
Supplement: Supplementary file 1 — Supporting file 1: mco270879‐sup‐0001‐SuppMat.pdf [file MCO2-7-e70879-s001.pdf]

Supplementary Material

Reprogramming Glial Cell Metabolism via a tRNA Fragment Preserves Vision in Retinal Neurodegeneration

Yuke Ji [1,2 #], Sha Liu [1 #], Ying Zhang [1 #], Junya Zhu [3 #], Chang Huang [4], Ya Zhao [5], Jiao Xia [5], Wan Mu [4 *], Jin Yao [1 *], Biao Yan [4, 5 *]

[1] Department of Ophthalmology and Optometry, The Affiliated Eye Hospital, Nanjing Medical University, Nanjing 210029, China
[2] Shenzhen Eye Hospital, Jinan University, Shenzhen 518000, China
[3] School of Medicine, Southeast University, Nanjing 210009, China
[4] Eye Institute and Department of Ophthalmology, Eye and ENT Hospital, Fudan University, Shanghai 200031, China
[5] Department of Ophthalmology, Shanghai General Hospital, Shanghai Jiao Tong University School of Medicine, Shanghai 200080, China

[#] These authors have contributed equally to this work.

[*] **Correspondence:**
yanbiao@sjtu.edu.cn (B.Y.)
jinyao1972@126.com (J.Y.)
muwanlab@163.com (W.M.)

Supplemental Figures

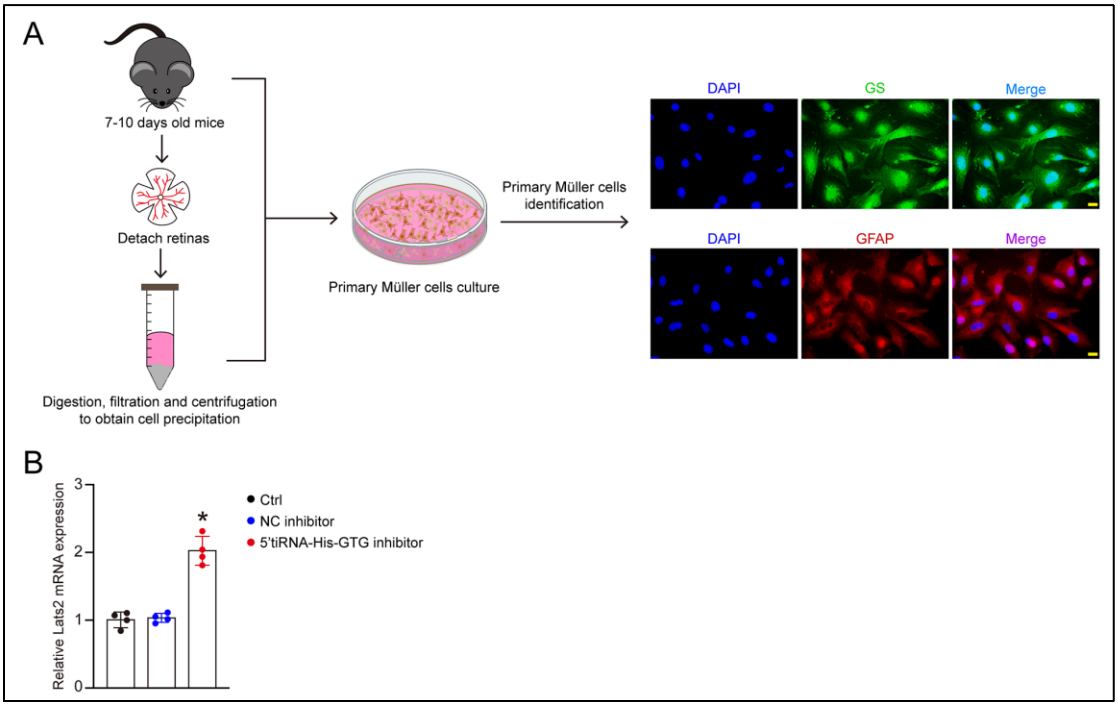

Figure S1: Isolation, Identification, and Transfection Efficiency Verification of Müller Cells

(A) Schematic illustration of primary Müller cell isolation and identification. Müller cells were identified by co-immunofluorescence staining for GS and GFAP. GS, green; GFAP, red; DAPI, blue. Scale bar, 20 μm. (B) Müller cells were transfected with a negative control (NC) inhibitor, 5′tiRNA-His-GTG inhibitor, or left untreated (Ctrl) for 24 h. The mRNA level of Lats2, a putative target of 5′tiRNA-His-GTG, was measured by qRT-PCR (n = 4; $*P < 0.05$; one-way ANOVA with Bonferroni post hoc test).

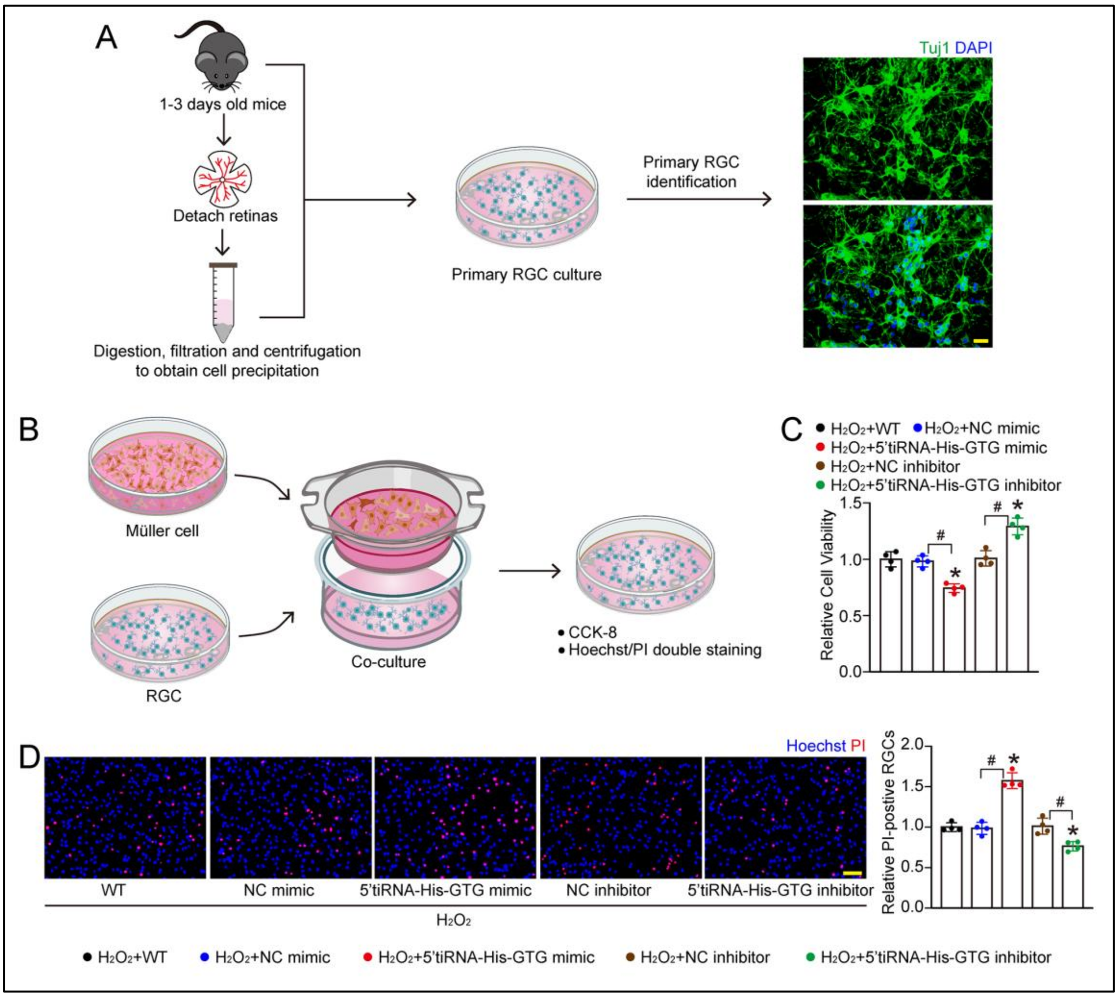

Figure S2: 5′tiRNA-His-GTG Indirectly Affects RGC Function in vitro

(A) Schematic illustration of primary RGC isolation and identification. RGCs were identified by immunofluorescence staining of Tuj1. Tuj1, green; DAPI, blue. Scale bar, 20 μm. (B) Schematic diagram of the co-culture system of RGCs and Müller cells. (C, D) RGCs were co-cultured with Müller cells (WT) or the corresponding transfected Müller cells and then subjected to H_2O_2 (200 μM) for 48 h. Cell viability was detected by CCK-8 assay (C, n = 4). Cell apoptosis was evaluated by Hoechst/PI double staining. PI, red; Hoechst, blue. Scale bar, 50 μm (D, n = 4). $^{*}P < 0.05$ versus WT; $^{\#}P < 0.05$ between the indicated groups; One-way ANOVA followed by Bonferroni post hoc test.

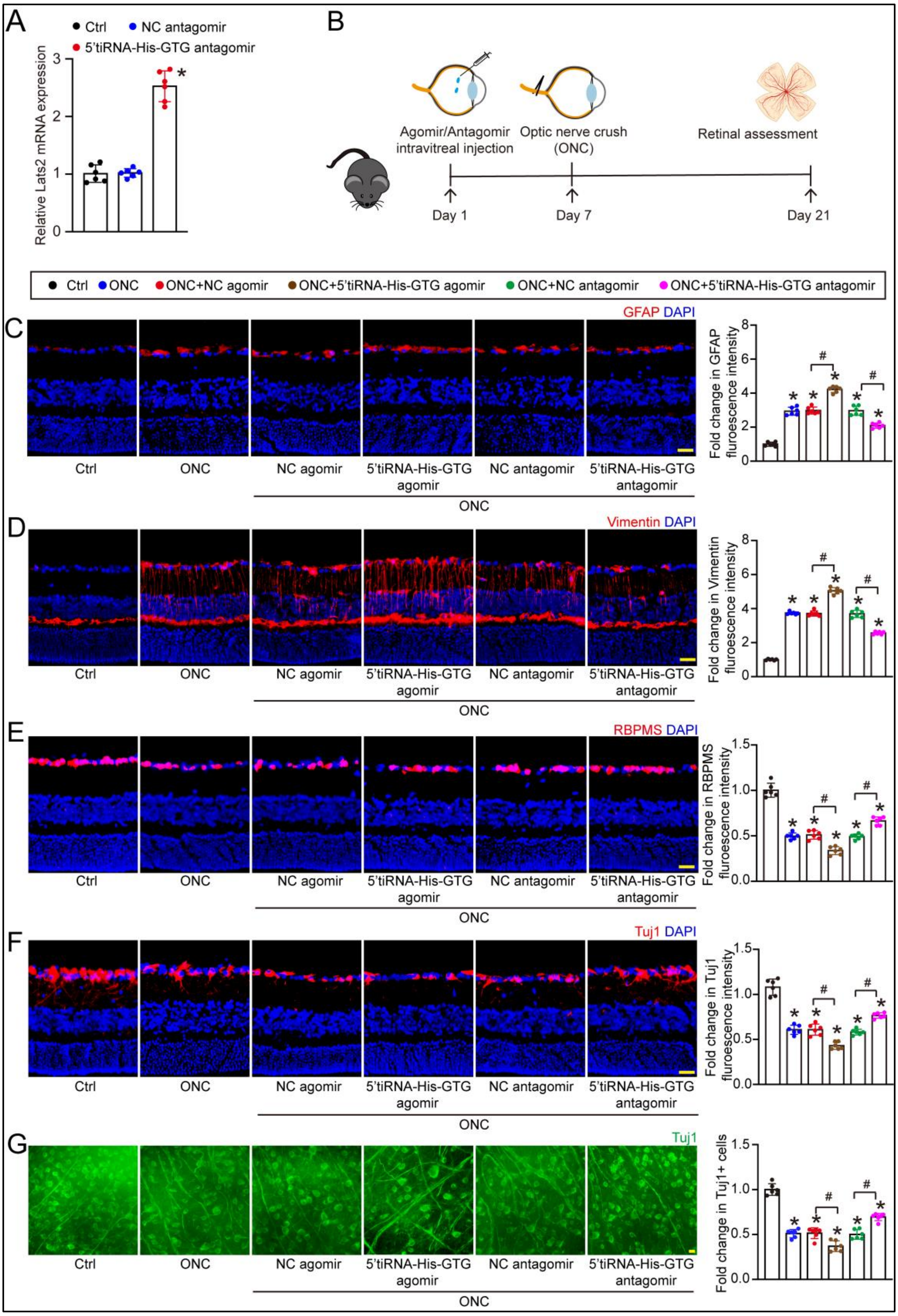

Figure S3: 5′tiRNA-His-GTG Regulates Retinal Neurodegeneration in vivo

(A) Eight-week-old male C57BL/6 mice received intravitreal injections of negative control (NC) antagomir or 5′tiRNA-His-GTG antagomir, while untreated mice served as controls (Ctrl). Retinal tissues were collected one week later, and the mRNA level of

Lats2, a validated target of 5′tiRNA-His-GTG, was detected by qRT-PCR (n = 6; *$P <$ 0.05; one-way ANOVA with Bonferroni post hoc test). (B) Schematic illustration of the experimental workflow for panels (C-G). (C, D) Eight-week-old C57BL/6 mice received intravitreal injections of NC agomir, 5′tiRNA-His-GTG agomir, NC antagomir, or 5′tiRNA-His-GTG antagomir. The left untreated mice were taken as the control (Ctrl) group. One week later, ONC was performed. Retinas were collected two weeks after ONC and subjected to immunofluorescence staining for GFAP (C) and Vimentin (D) to assess reactive gliosis (n = 6). Nuclei were counterstained with DAPI (blue). Scale bars, 50 μm. (E, F) Immunofluorescence staining for RBPMS (E) and Tuj1 (F) was performed to evaluate RGC survival (n = 6). Nuclei were counterstained with DAPI (blue). Scale bars, 50 μm. (G) Retinal whole-mount staining with Tuj1 was used to visualize and quantify surviving RGCs (n = 6). Scale bar, 20 μm. *$P < 0.05$ versus Ctrl; $^{\#}P < 0.05$ between the indicated group; One-way ANOVA with Bonferroni post hoc test.

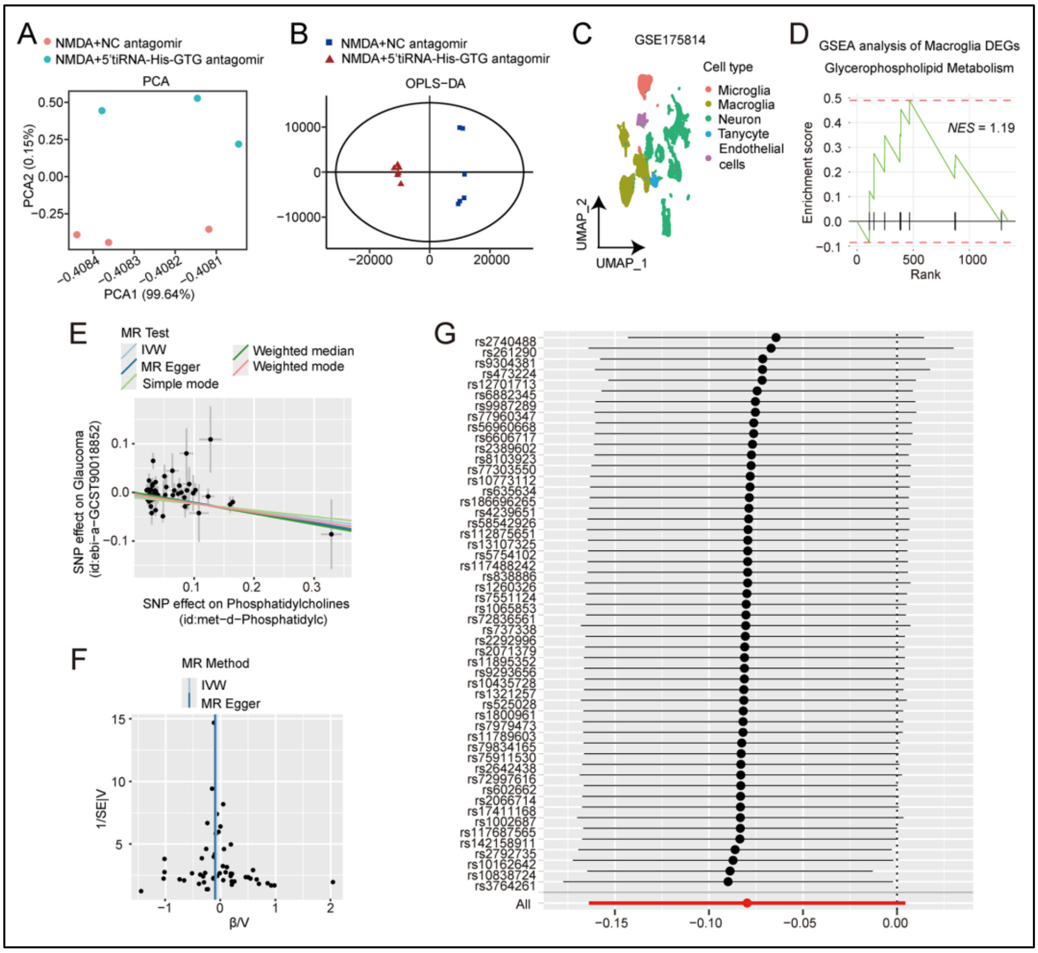

Figure S4: Glycerophospholipid Metabolism is Altered in Neurodegenerative Diseases

(A, B) Eight-week-old male C57BL/6 mice received intravitreal injections of 5′tiRNA-His-GTG antagomir or negative control (NC) antagomir. One week later, NMDA (20 mM, 1.5 μl per eye) was administered via intravitreal injection. Retinas were collected one week after NMDA treatment. Principal component analysis (PCA) of RNA-seq data (n = 3). Samples from NMDA + NC antagomir and NMDA + 5′tiRNA-His-GTG antagomir groups are clearly separated, indicating distinct transcriptomic profiles. The x- and y-axes represent the variance explained by PC1 and PC2, respectively (A). Orthogonal partial least squares discriminant analysis (OPLS-DA) score plot of metabolomic profiles comparing NMDA + NC antagomir and NMDA + 5′tiRNA-His-GTG antagomir groups (n = 6), showing clear group separation (B). (C, D) Analysis of single-cell RNA-seq data from human Alzheimer’s disease (AD) brains (GSE175814). Uniform manifold approximation and projection (UMAP) visualization of major cell populations, color-coded by cluster identity (C). Gene set enrichment analysis (GSEA) of glycerophospholipid metabolism pathway in glial cells from AD and control brains, indicating enrichment of this pathway in disease-associated glial populations (D). (E-G) Mendelian randomization (MR) analysis evaluating the association between phosphatidylcholine (PC) levels and glaucoma risk. Scatter plot (E), funnel plot (F), and leave-one-out plot (G) illustrating MR analysis of PC-associated variants and glaucoma risk.

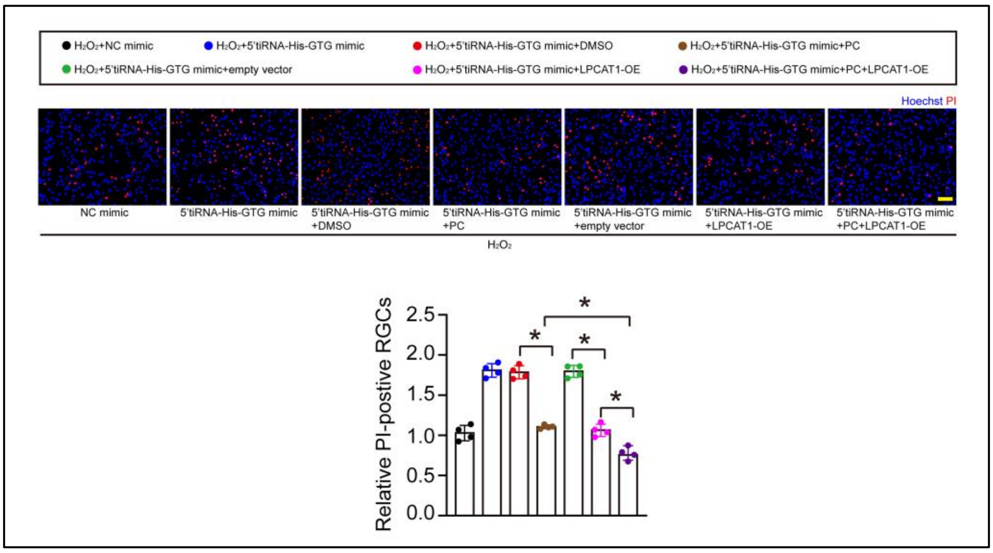

Figure S5: 5′tiRNA-His-GTG Indirectly Affects RGC Function via LPCAT1-Phosphatidylcholine Axis in vitro

RGCs were co-cultured with Müller cells transfected with negative control (NC) mimic, 5′tiRNA-His-GTG mimic, 5′tiRNA-His-GTG mimic plus phosphatidylcholine (PC, 50 μM) or DMSO, or 5′tiRNA-His-GTG mimic plus empty vector or LPCAT1-overexpression (LPCAT1-OE). After co-culture, cells were exposed to H_2O_2 for 48 h. Cell apoptosis was assessed by Hoechst/PI double staining. PI, red; Hoechst, blue; *$P < 0.05$ between indicated groups; One-way ANOVA followed by Bonferroni post hoc test.

Supplemental Materials and Methods

Mice Breeding

Eight-week-old male C57BL/6 mice were purchased from Hangzhou Ziyuan Laboratory Animal Technology (Zhejiang, China). They were maintained under specific pathogen-free conditions at 25°C with a 12 h light/12 h dark cycle. All procedures were conducted in accordance with institutional guidelines for the care and use of laboratory animals and were approved by the Animal Ethics Committee of Nanjing Medical University.

RNA Extraction and Quantitative Reverse Transcription-PCR (qRT-PCR) Assay

Total RNA was extracted from cells or retinas using TRIzol reagent (15596026, Life Technologies, USA) and quantified using a spectrophotometer (Agilent, USA). cDNA was synthesized using the rtStar™ First-Strand cDNA Synthesis Kit (AS-FS-003, Arraystar, USA) or the SuperScript IV First-Strand Synthesis System (18091050, Thermo Fisher Scientific, USA). Quantitative PCR was performed using SYBR Green Master Mix (100029284, Thermo Fisher Scientific, USA) on a PikoReal Real-Time PCR System (Thermo Fisher Scientific, USA). All reactions were conducted in triplicate. Relative gene expression levels were calculated using the $2^{-\Delta\Delta Ct}$ method. tiRNA levels were quantified using a stem-loop reverse transcription PCR method. Primer sequences are listed in Table S2.

Intravitreal Injection

Agomirs and antagomirs for 5′tiRNA-His-GTG, along with corresponding negative controls, were purchased from RiboBio (Guangzhou, China). The mice were

subjected to anesthesia before proceeding with the experimental treatment. A volume of 1.5 μl of either 5'tiRNA-His-GTG agomir or its corresponding 5'tiRNA-His-GTG antagomir was injected into the vitreous cavity. As control, an equivalent amount of negative control (NC) agomir/antagomir was injected intravitreally. The intravitreal injections were performed using a microinjector, which was equipped with a 33-gauge needle to ensure minimal trauma to the eye. The needle was accurately positioned to reach the vitreous, specifically 1 mm posterior to the corneal limbus, ensuring the delivery of the treatment to the intended location. Post-injection, topical antibiotics were applied to reduce the risk of infection. The injection volume was within a range that does not significantly affect intraocular pressure.

Retinal Flat-Mount Staining

Eyeballs were fixed in 4% paraformaldehyde (PFA) for 30 min at room temperature. Retinas were carefully dissected from the sclera, flattened by making four radial incisions, and mounted on glass slides. Tissues were permeabilized and blocked in PBS containing 1% Triton X-100 and 5% BSA for 45 min at 37°C. Samples were incubated with Tuj1 antibody overnight at 4°C, followed by incubation with Alexa Fluor 488-conjugated goat anti-rabbit IgG (A-11008, Thermo Fisher Scientific, USA) for 2 h at room temperature. Images were acquired using an Olympus IX-73 microscope.

Cell Transfection

Mimics and inhibitors for 5′tiRNA-His-GTG, along with corresponding negative controls, were purchased from RiboBio (Guangzhou, China). Müller cells were seeded in 24-well plates and grown to 80-85% confluence. Cells were transfected with 50 nM

oligonucleotides using Lipofectamine 3000 (L3000150, Life Technologies, USA). After 4-6 h, the medium was replaced with fresh DMEM/F12 supplemented with 10% FBS. Cells were then cultured at 37°C for an additional 24 h before further experiments.

Cell Counting Kit-8 Assay

After transfection, Müller cells were either left untreated or exposed to H_2O_2 at 200 μM for 48 h. Subsequently, CCK-8 (10 μg/mL, C0038, Beyotime, China) was added to each well for 1 h. Cell viability was analyzed in a Filter Max F5 Microplate Reader (Molecular Devices, USA) at 450 nm.

EdU Incorporation Assay

The BeyoClickTM EdU detection kit (C0071S, Beyotime, China) was used to detect cell proliferation. Treated Müller cells were grown in DMEM-F12 containing EdU at 37°C. Three hours later, the samples were fixed with 4% PFA for 15 min and permeabilized with 0.3% Triton-X100 for 15 min. Afterward, click reaction cocktail was incubated with cells for 30 min at room temperature in dark. DAPI was used to counterstain nuclei. EdU-positive cells were counted and imaged using an Olympus IX-73 microscope and analyzed using ImageJ (NIH, USA) software.

Propidium Iodide (PI) Staining

Treated Müller cells were flushed three times in warm PBS, followed by co-staining with 10 μM of Calcein-AM (22002, AAT Bioquest, USA) and 10 μM of PI (C2015S, Beyotime, China) for 15 min. Similarly, RGCs were stained with 3 μM of Hoechst 33342 (C1022, Beyotime, China) and 10 μM of PI for 15 min. Cells were washed with the medium and imaged using an Olympus IX-73 microscope.

Cell Co-Culture Assay

A transwell-based co-culture system was used to determine the effects of Müller cells on RGC function. Müller cells and RGCs were cultured in upper and lower chambers, respectively, separated by a 0.4 μm pore membrane. RGCs (2.5×10^5 cells/well) were seeded in 24-well plates. After 6-8 h, the medium was replaced with DMEM/F12 containing 10% FBS. Treated Müller cells (1.0×10^5 cells/well) were then added to the upper chamber and co-cultured with RGCs for 24 h at 37°C in a humidified incubator with 5% CO_2.

Terminal Deoxynucleotidyl Transferase dUTP Nick End Labeling (TUNEL) Assay

Apoptosis of Müller cells was assessed using the one step TUNEL apoptosis assay kit (C1088, Beyotime, China). Cells were washed three times with warm PBS and fixed in 4% paraformaldehyde for 30 min. Samples were then incubated with TUNEL reaction buffer for 1 h according to the manufacturer’s instructions. TUNEL-positive cells were analyzed using an Olympus IX-73 fluorescence microscope.

Western Blot

Samples were lysed in RIPA buffer (P0013B, Beyotime, China). After centrifugation at 12,000 × *g* for 20 min at 4°C, the supernatants were collected. Protein concentrations were determined using a BCA Protein Assay Kit (P0010, Beyotime, China). Protein samples were boiled for 10 min, separated by 10% SDS-PAGE, and transferred to PVDF membranes (IPVH00010, Millipore, USA). Membranes were blocked with 5% non-fat milk in TBST for 1 h, incubated with primary antibodies overnight at 4°C, and then with HRP-conjugated secondary antibodies for 2 h at room

temperature. Protein bands were visualized using a high-sensitivity ECL chemiluminescence kit (G2020-500, Servicebio, China).

Statistical Analysis

Data were analyzed using GraphPad Prism 8 (GraphPad Software, USA) and are presented as mean ± SEM. For normally distributed data with equal variance, Student's *t*-test was used for comparisons between two groups. For comparisons among multiple groups, one-way ANOVA followed by Bonferroni post hoc test was performed. When data did not meet normality or equal variance assumptions, non-parametric tests were applied. $P < 0.05$ was considered statistically significant.

Table S1. Antibodies used in this study

Antibodies	Source	Identifier	Dilution	Application
RBPMS	Abcam	ab152101	1:500	Immunofluorescence
Tuj1	Abcam	ab18207	1:500	Immunofluorescence
GFAP	Abcam	ab68428	1:500	Immunofluorescence
Vimentin	Abcam	ab92547	1:1000	Immunofluorescence
Angiogenin	Proteintech	18302-1-AP	1:1000	Western blot
LPCAT1	Proteintech	16112-1-AP	1:2000	Western blot
LPCAT2	Proteintech	15082-1-AP	1:5000	Western blot
LPCAT3	Abmart	PK53132S	1:2000	Western blot
LPCAT4	Proteintech	17905-1-AP	1:2000	Western blot
